# Multi-Modal Assessment of Cerebral Hemodynamics in Resuscitated Out-of-Hospital Cardiac Arrest Patients: A Case-Series

**DOI:** 10.3390/life14091067

**Published:** 2024-08-26

**Authors:** Shir Lynn Lim, May Zin Myint, Kai Lee Woo, Elaine Young Heng Chee, Chiew Sie Hong, Erta Beqiri, Peter Smielewski, Marcus Eng Hock Ong, Vijay Kumar Sharma

**Affiliations:** 1 Department of Cardiology, National University Heart Centre, Singapore 119228, Singapore; kai_lee_woo@nuhs.edu.sg; 2 Yong Loo Lin School of Medicine, National University of Singapore, Singapore 117597, Singapore; mdcvks@nus.edu.sg; 3 Pre-Hospital and Emergency Research Centre, Duke-NUS Medical School, Singapore 169857, Singapore; 4 Division of Neurology, Department of Medicine, National University Hospital, Singapore 119074, Singapore; may.myint@mohh.com.sg (M.Z.M.); young_heng_chee@nuhs.edu.sg (E.Y.H.C.); chiew_sie_hong@nuhs.edu.sg (C.S.H.); 5 Brain Physics Laboratory, Division of Neurosurgery, Department of Clinical Neurosciences, University of Cambridge, Cambridge CB2 1TN, UK; vb391@cam.ac.uk (E.B.); ps10011@cam.ac.uk (P.S.); 6 Department of Emergency Medicine, Singapore General Hospital, Singapore 169608, Singapore; marcus.ong@duke-nus.edu.sg; 7 Health Services and Systems Research, Duke-NUS Medical School, Singapore 169857, Singapore

**Keywords:** cerebral hemodynamics, cerebral autoregulation, resuscitated out-of-hospital cardiac arrest, transcranial Doppler ultrasound, near-infrared spectroscopy

## Abstract

We assessed the feasibility of concurrent monitoring of cerebral hemodynamics in adult, comatose out-of-hospital cardiac arrest (OHCA) patients admitted to the National University Heart Centre Singapore from October 2021 to August 2023. Patients underwent continuous near-infrared spectroscopy (NIRS) monitoring in the first 72 h after return of spontaneous circulation (ROSC) and 30-min transcranial Doppler ultrasound (TCD) monitoring at least once. With constant mechanical ventilatory settings and continuous electrocardiographic, pulse oximeter and end-tidal carbon dioxide monitoring, blood pressure was manipulated via vasopressors and cerebral autoregulation assessed by measuring changes in regional cerebral oxygenation (NIRS) and cerebral blood flow velocities (TCD) in response to changes in mean arterial pressure. The primary outcome was neurological recovery at hospital discharge. Amongst the first 16 patients (median age 61, 94% males), we observed four unique patterns: preserved cerebral autoregulation, loss of cerebral autoregulation, cardio-cerebral asynchrony and cerebral circulatory arrest. Patients with preserved cerebral autoregulation had lower levels of neuro-injury biomarkers (neurofilaments light and heavy) and the majority (86%) were discharged with good neurological recovery. Multi-modal assessment of cerebral hemodynamics after OHCA is feasible and derived patterns correlated with neurological outcomes. The between- and within-patient heterogeneity in cerebral hemodynamics calls for more research on individualized treatment strategies.

## 1. Introduction

In cardiac arrest, there are complex and bidirectional interactions between the cardiovascular and central nervous systems, which form part of the heart-brain axis. The initial global ischemia followed by ischemia-reperfusion after return of spontaneous circulation (ROSC) lead to energy failure, excitotoxicity, oxidative stress and systemic inflammation. These contribute to the brain injury seen in resuscitated out-of-hospital cardiac arrest (OHCA) patients, which accounts for two-thirds of mortality [1]. Key to neuroprotection is optimizing cerebral blood flow, which is determined by the mean arterial blood pressure (MAP) and local vascular resistance. The optimal blood pressure (BP) in the early (first 12 h) and intermediate (12–72 h) post-return of spontaneous circulation (ROSC) periods is unclear. Targeting a MAP of 65 mmHg, in accordance with international guidelines [2,3], has been associated with a profound decrease in cerebral oxygenation during the critical 6–12 h of ICU stay [4,5]. Yet, randomized controlled trials (RCT) and meta-analysis comparing different MAP targets failed to detect differences in survival or favorable neurological outcomes with higher MAP targets [6,7,8,9,10]. 

The challenges faced in elucidating MAP targets after ROSC may be explained by: (a) Phasic variation in cerebral blood flow is observed after ROSC, characterized by early but brief hyperemia (0–20 min after ROSC), followed by hypoperfusion (20 min–12 h after ROSC), and finally restoration of normal flow in the final phase (12–72 h after ROSC) [11]. (b) Cerebral autoregulation may be completely lost or impaired for a variable period in some patients, with a narrowed and right-shifted intact zone [12,13]. The range of MAP in which autoregulation could maintain sufficient cerebral perfusion appears to be heterogenous among individuals. Taken together, there is within- and between-patient heterogeneity in the influence of MAP on cerebral blood flow, depending on the phase of post-resuscitation period and individual thresholds for cerebral autoregulation, respectively. 

Prior studies on neuromonitoring in resuscitated OHCA patients were limited by the use of a single neuromonitoring modality; this was commonly the near-infrared spectroscopy (NIRS) [14,15,16] and, less commonly, transcranial Doppler ultrasound (TCD) [12]. While NIRS provides real-time information about oxygenation in the superficial cortical layers of the brain underlying the sensor pads, TCD demonstrates blood flow velocities in deeper cerebral arteries as well as flow resistance, reflecting the status of the distal cerebral perfusion. Both NIRS and TCD are non-invasive, portable and may be used concurrently on the same patient, thus providing complementary information on person- and time-specific microvascular and macrovascular cerebral blood flow. Here, we report a case-series in which we evaluated the feasibility of multi-modal monitoring of cerebral hemodynamics using NIRS and TCD in resuscitated OHCA patients and explored the associations between cerebral autoregulation (in response to pharmacological manipulations of MAP) with neurological recovery.

## 2. Materials and Methods

### 2.1. Study Design and Population

This neuromonitoring sub-study from the prospective observational Early Neuroprognostication For Out-of-Hospital Cardiac Arrest (ENFORCE) study was conducted in the National University Hospital, an academic medical center in Singapore with 1160 beds. The ENFORCE study, which aimed to identify promising candidate biomarkers for early neuroprognostication after cardiac arrest, recruited adult (≥21 years), comatose, resuscitated OHCA patients of presumed cardiac etiology who were admitted to the Coronary Care Unit (CCU). 

### 2.2. Study Activities

All resuscitated OHCA patients admitted to CCU were managed according to the institutional protocol which included: (1) Emergency coronary angiogram, and revascularization if required, in patients deemed to be at high risk of acute coronary occlusion. (2) Targeted temperature management using surface cooling with Blanketrol II to 33 Celcius. (3) Mechanical ventilation to achieve normal levels of partial arterial oxygen (PaO_2_) and carbon dioxide pressures (PaCO_2_). (4) Standardized multi-modal neuroprognostication after 96 h post-ROSC.

### 2.3. Biomarkers

Designated research coordinators collected blood samples, 10 mls each time, from enrolled patients within 4 h of last sustained ROSC, and at 24 and 72 h after ROSC. Blood was processed within one hour. Plasma was separated by centrifugation at 3500× *g* for 10 min at 4 °C and stored at −80 °C until batch analysis of neurofilament light (NF-L), neurofilament heavy (NF-H) and soluble urokinase plasminogen activator receptor (suPAR). NF-L and NF-H were measured on the fully automated ELLA immunoassay system (ProteinSimple; Bio-Techne, Minneapolis, MN, USA), while suPAR was measured by microplate-based sandwich ELISA (ViroGates, Birkeroed, Denmark; suPARnostic^®^ AUTO Flex ELISA, Code No. E001) on the Enspire Multimode Microplate Reader (Perkin Elmer, Waltham, MA, USA). 

### 2.4. Neuromonitoring and Data Recording

From March 2019, all resuscitated OHCA patients admitted to CCU had continuous monitoring of regional cerebral oxygenation (rSO_2_) using the INVOS cerebral oximeter (Covidien, Dublin, Ireland) as part of post-ROSC bundle of care. Sensors were applied bilaterally to the frontotemporal areas as soon as the patients were admitted and kept on till 72 h post-ROSC. Hourly left and right rSO_2_ data were recorded together with hemodynamic parameters. 

From October 2021, TCD examination was performed at least once post-ROSC while rSO_2_ monitoring was in progress; this was performed at the earliest opportunity, preferably within 24 h. Using Dolphin 4D Viasonix, TCD spectra were obtained from both middle cerebral arteries (MCA) using the standard Spencer’s headframe. TCD 2-MHz monitoring probes were mounted on the headframe and directed to slightly anterior and superior orientation through the respective transtemporal acoustic windows to obtain the strongest flow signals from the ipsilateral MCAs from a depth of 50–60 mm (representing the horizontal M1 segment). TCD monitoring was performed continuously for a duration of 30–45 min for each assessment.

### 2.5. Assessment of Cerebral Autoregulation [17,18,19]

Cerebral autoregulation was assessed by measuring the changes in MCA blood flow velocities (CBFV) and rSO_2_, in response to changes in MAP. This was performed with constant (unchanged) mechanical ventilatory settings and continuous electrocardiographic (ECG), pulse oximeter and end-tidal carbon dioxide monitoring. BP was manipulated in a stepwise manner through increasing and then decreasing the dose of vasopressors to achieve MAPs ranging from 50 mmHg to 110 mmHg. 

rSO_2_ was continuously monitored during this same period. High resolution data of rSO_2_ (every second) and arterial line data (125 Hz) were collected using the ICM+ software version 9.1 (Cambridge Enterprise, Cambridge, UK). The signals were streamed from the monitors to a laptop where the ICM+ software was running, providing data synchronization. The same software was used for data processing. Arterial BP artefacts were automatically removed using the absence of pulse detection and by rejecting non-physiological values. Ten-second averages of rSO_2_ and MAP were calculated. Cerebral oxygenation index (CO_x_) was assessed as a moving Pearson correlation coefficient (a value ranging from −1 to +1) between 30 consecutive, 10-s averaged values of MAP and corresponding rSO_2_ signals. A positive CO_x_ value of greater than or equal to 0.3 was considered to indicate dysfunctional autoregulation [20,21]. TCD spectra were recorded continuously for 5 min to obtain the baseline blood flow characteristics, followed by another 30 to 45 min during blood pressure manipulation. The mean flow index (Mx) values were calculated for assessing dynamic cerebral autoregulation. Briefly, the raw recording was averaged into blocks, which were then split into epochs, a Pearson’s correlation coefficient was calculated for every epoch, and the average of the correlation coefficient from the epochs generated the Mx. While an increase in Mx indicated poor dynamic cerebral autoregulation, a decreased value suggested better dynamic cerebral autoregulation. Additionally, qualitative cerebral autoregulation was assessed by observing the changes in TCD-derived CBFV and pulsatility index (PI), in real-time, in response to the pharmacological manipulation of MAP. In response to the elevation of MAP, a transient increase in CBFV followed by a return to baseline values within a couple of minutes and associated with an increase in PI (representing vasoconstriction) suggested an intact dynamic cerebral autoregulation. Similarly, a transient decrease in CBFV during reduction of MAP and associated with decrease in PI (representing vasodilatation) also represented intact cerebral autoregulation.

Where there was disagreement on the state of cerebral autoregulation between CO_x_ and Mx, autoregulatory status based on Mx was used, as CBFV is a closer surrogate of CBF than rSO_2_ is. 

### 2.6. Clinical Data and Outcomes

We collected data on patient demographics and medical co-morbidities, as well as OHCA characteristics, management and outcomes. Data were extracted from emergency dispatch records, ambulance case notes, ED and in-hospital records. In-hospital records included cardiac catheterization, echocardiography and electrocardiogram reports, and medical and nursing notes. Results of NF-L, NF-H and suPAR were obtained from the Cardiovascular Research Institute Biomarker Laboratory. The primary outcome was survival to hospital discharge with good neurological recovery, where good neurological recovery was defined as having a Cerebral Performance Category (CPC) of 1 or 2 [22]. 

## 3. Results

Between October 2021 and August 2023, 16 patients (median age 61 years, 94% males) had combined neuromonitoring with both cerebral oximeter and TCD (Table 1). This was aborted in one patient who had hemodynamically unstable ventricular tachycardia during TCD. Of these patients, seven had preserved cerebral autoregulation, five had loss of cerebral autoregulation, two had cerebral circulatory arrest and one had cardio-cerebral asynchrony. We observed changing patterns of cerebral autoregulation in one patient, where it evolved from impaired autoregulation on D1 to normalization by D2; this patient went on to have good neurological recovery. Here we present four patients, representing the four unique patterns of cerebral autoregulation observed in our study. 

### 3.1. Patient A

A 65-year-old Chinese man with hypertension suffered an OHCA, which was preceded by chest pain. Bystander cardiopulmonary resuscitation (CPR) was performed by his son. The first recorded rhythm was ventricular fibrillation (VF) but deteriorated to pulseless electrical activity (PEA) subsequently. Sustained return of spontaneous circulation (ROSC) was achieved after 91 min of resuscitation, in the Emergency Department (ED). Post-ROSC ECG showed ST-elevations in the inferior leads; emergency coronary angiogram identified a thrombotic occlusion of the distal obtuse marginal branch of the left circumflex artery which was intervened. 

**Figure 1:**
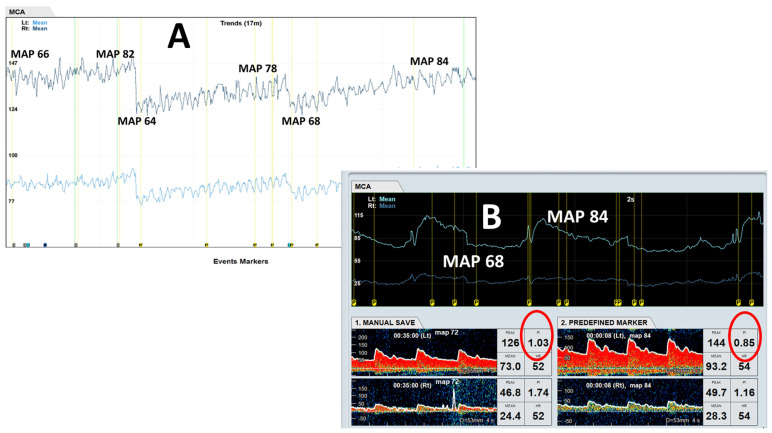
Loss of cerebral autoregulation. (**A**) shows the traces for trends of mean flow velocities for bilateral MCAs during continuous TCD monitoring shown. Note the linear relationship between mean flow velocities of bilateral MCAs and MAP, suggestive of loss of cerebral autoregulation. Additionally, (**B**) shows the Doppler spectra from both MCAs, which showed the paradoxical decrease in flow resistance represented by decreasing PI (PI from 1.03 to 0.85) with increasing MAP (68 to 84 mmHg). Abbreviations: MCA, middle cerebral artery; TCD, transcranial Doppler; MAP, mean arterial pressure; PI, pulsatility index.

**Figure 2:**
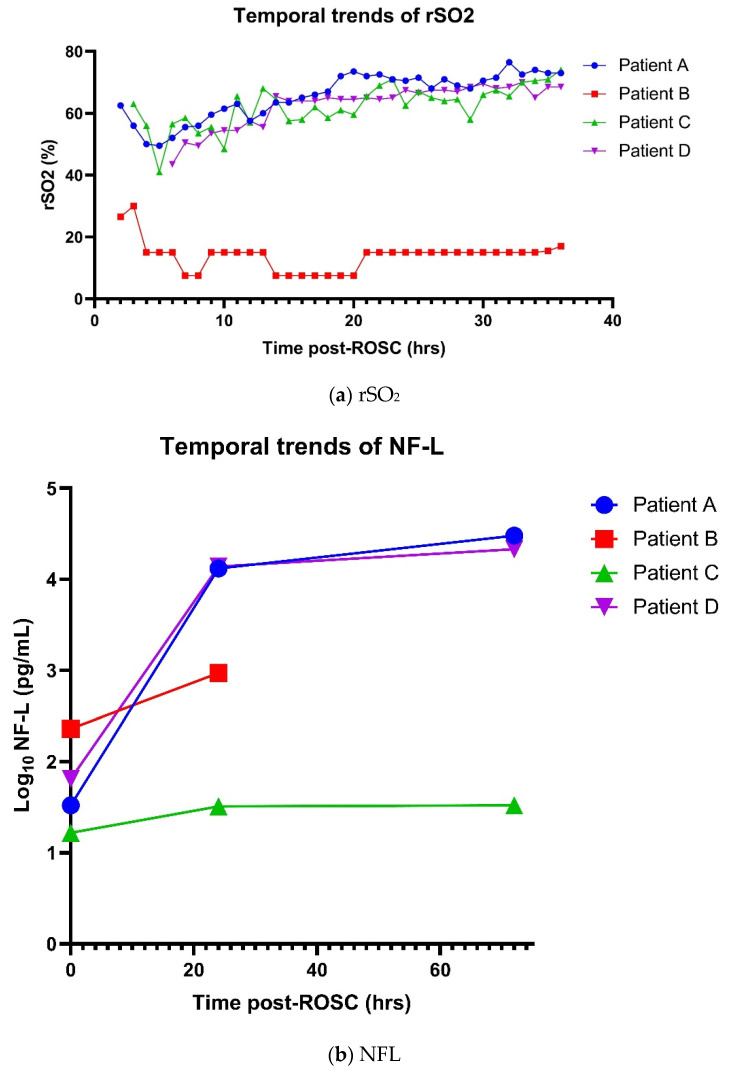

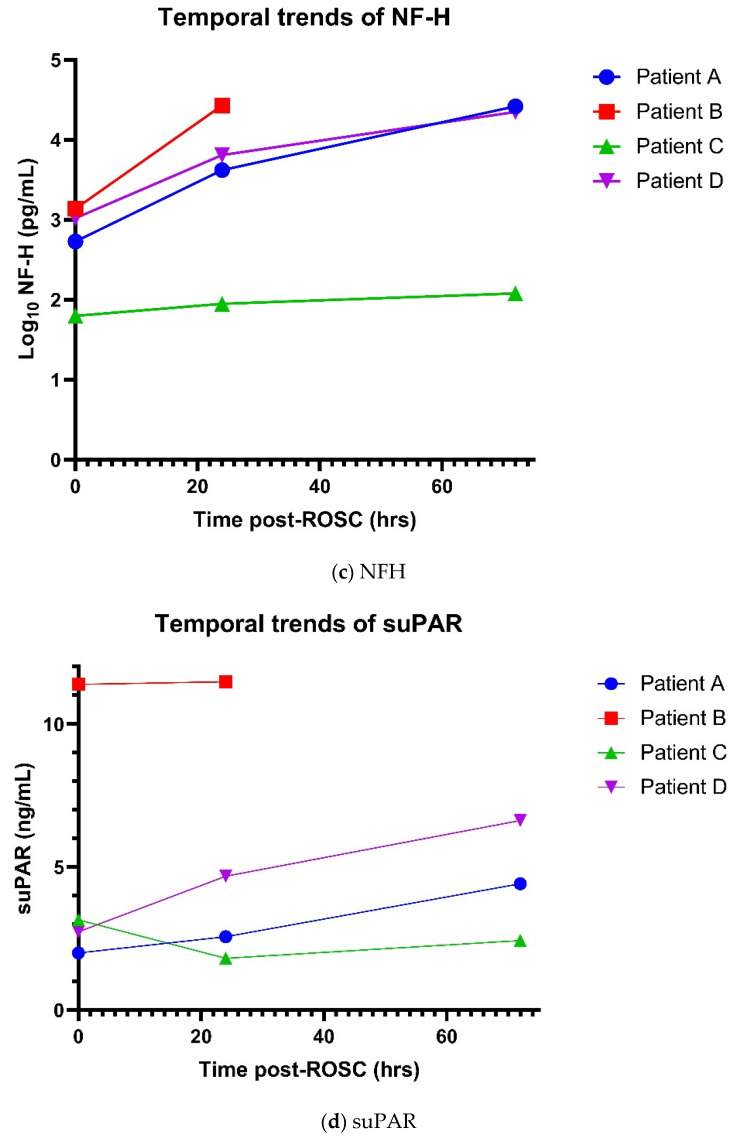
Temporal trends of rSO_2_ and neuro-injury biomarkers post-ROSC. Figure 2 shows the temporal trends of (**a**) rSO_2_, (**b**) NFL, (**c**) NFH and (**d**) suPAR for Patients A to D. Hourly right and left rSO_2_ were recorded, and the average of right and left rSO_2_ values were plotted for the first 36 h after ROSC. Neuro-injury biomarkers were measured as soon as possible after ROSC, and at 24 h and 72 h after ROSC. Abbreviations: rSO_2_, regional cerebral tissue oxygenation; ROSC, return of spontaneous circulation; NFL, neurofilament light; NFH, neurofilament heavy; suPAR, soluble urokinase plasminogen activator receptor.

Bedside TCD assessment and evaluation of cerebral autoregulation was performed daily during the first 4 consecutive days of admission (Figure 1A). Findings were consistent across the 4 days where a linear relationship between mean CBFV and MAP was noted, indicating the loss of cerebral autoregulation. Further, there was increased hyperemia (as evidenced by decreased in PI) with increasing MAP (Figure 1B). Average of left and right rSO_2_ showed an initial dip after 2 h post-ROSC before recovering from 6 h post-ROSC onwards to finally plateauing after 20 h post-ROSC, consistent with phasic variations in cerebral blood flow after cardiac arrest (Figure 2a). Levels of NF-L, NF-H and suPAR were elevated immediately post-ROSC and increased exponentially over 72 h (Figure 2b–d). 

**Figure 3:**
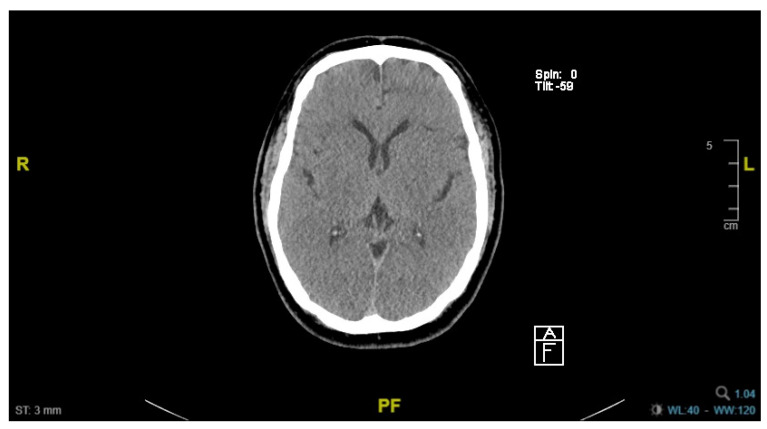
Computed tomography (CT) of brain. CT Brain performed on day 6 of admission showed complete loss of grey-white matter differentiation and generalized cerebral swelling with effacement of the sulci.

Clinical examination on day 6 of admission (D6) showed poor Glasgow Coma Scale (GCS) despite intact brainstem reflexes. Computed tomography of the brain (CT Brain) showed complete loss of grey-white matter differentiation within the supra- and infra-tentorial brain, and generalized cerebral swelling with effacement of the sulci, findings consistent with global hypoxic ischemic injury (Figure 3). A joint decision was made between managing clinical team and family for withdrawal of life-sustaining therapies (WLST) given little chances of meaningful neurological recovery. 

### 3.2. Patient B

A 69-year-old Chinese woman, with known co-morbidities of hypertension, dyslipidemia, diabetes mellitus and end-stage renal failure on regular hemodialysis, was admitted for a witnessed OHCA at home. Immediate bystander CPR was performed by her son. The initial rhythm was asystole, and that converted to VF subsequently. Sustained ROSC was achieved after 58 min of resuscitation; post-ROSC GCS was 3 and ECG showed widespread ST-depressions with ST-elevation in aVR. Consistent with ECG findings, emergency coronary angiogram showed severe and diffuse triple vessel disease; no coronary revascularization was done in view of lack of culprit lesion. 

**Figure 4:**
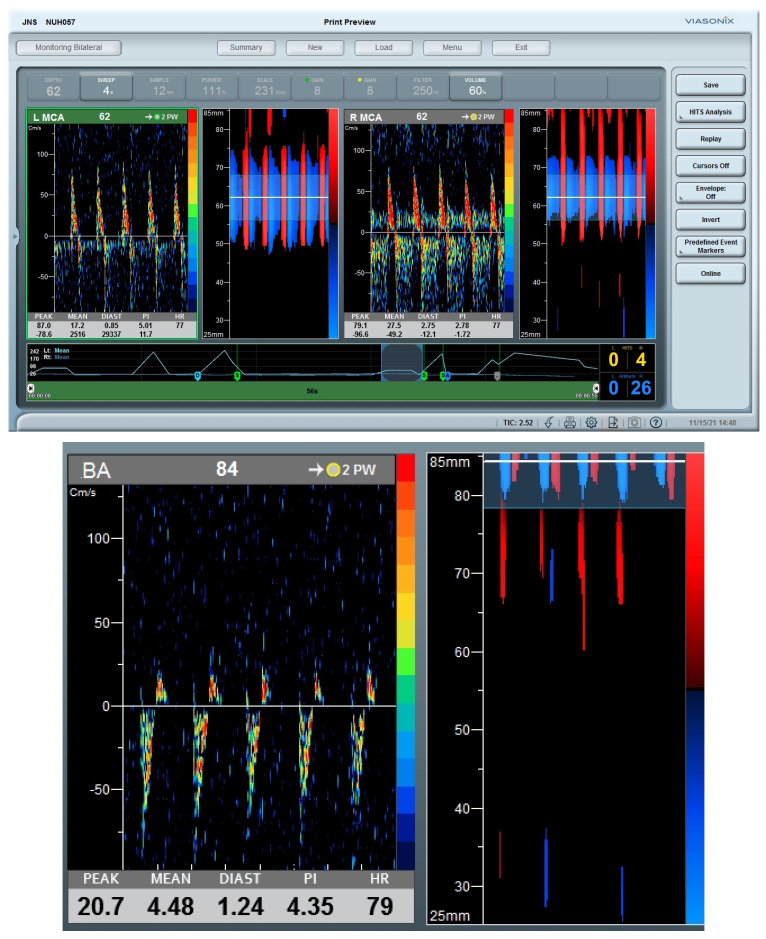
Cerebral circulatory arrest. Both MCAs (**upper panel**) and basilar artery (**lower panel**) demonstrate alternating (reverberating) Doppler spectra, suggestive of cerebral circulatory arrest. Abbreviations: MCA, middle cerebral artery.

TCD monitoring performed within 10 h of ROSC showed reverberating Doppler spectra in both ICA siphons and basilar artery, suggestive of cerebral circulatory arrest (Figure 4). No assessment of dynamic cerebral autoregulation was performed thereafter. Bilateral rSO_2_ readings were persistently below 20% after 6 h post-ROSC (Figure 2). Levels of NF-L, NF-H and suPAR were markedly elevated on admission, with 4-fold and 20-fold increases observed after 24 h in NF-L and NF-H (Figure 2). There were fixed and dilated pupils, and absent reflexes. In view of poor neurological prognosis, we did not attempt further invasive procedures including insertion of intra-arterial balloon pump (IABP); life-sustaining therapies were withdrawn and she passed away on D3 of admission. 

### 3.3. Patient C 

A 45-year-old Indian man, with known cardiovascular risk factors of chronic smoking, hypertension and dyslipidemia, suffered an OHCA at work. No bystander CPR was administered. The initial rhythm recorded by EMS was VF and he received 3 attempts at direct current cardioversion. There was ROSC en route to the hospital with a total downtime of 17 min; he remained comatose. Emergency coronary angiogram showed hemodynamically significant stenosis in the proximal left anterior descending artery which was stented. 

**Figure 5:**
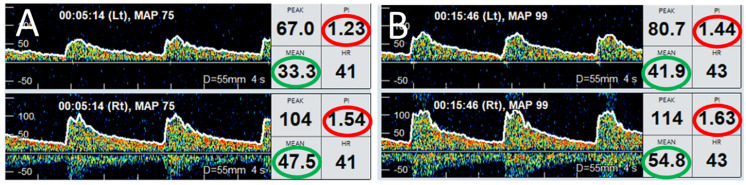
Preserved cerebral autoregulation. TCD monitoring performed on a 46-year-old man resuscitated after OHCA, on day 2 post-ROSC. (**A**) shows Doppler spectra from left (upper trace) and right (lower trace) MCA when MAP was 75 mmHg. (**B**) shows the Doppler spectra from both MCAs after MAP increased to 99 mmHg. Note that the MFV (within green circles) in left (33.3 cm/s) and right (47.5 cm/s) in panel A increased to 41.9 cm/s and 54.8 cm/s, respectively. The increase in MFV was accompanied by respective increases in PI values (within red circles), suggesting normal cerebral autoregulation. Abbreviations: OHCA, out-of-hospital cardiac arrest; TCD, transcranial Doppler ultrasound; ROSC, return of spontaneous circulation; MCA, middle cerebral artery; MAP, mean arterial pressure; MFV, mean flow velocity; PI, pulsatility index.

**Figure 6:**
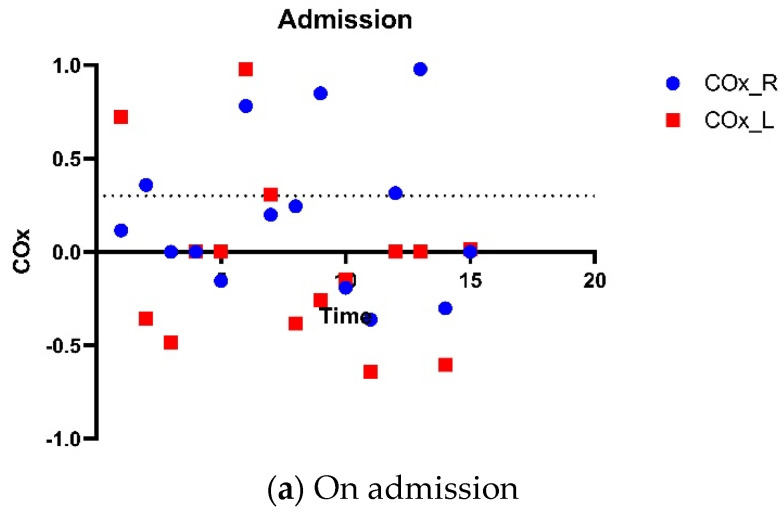

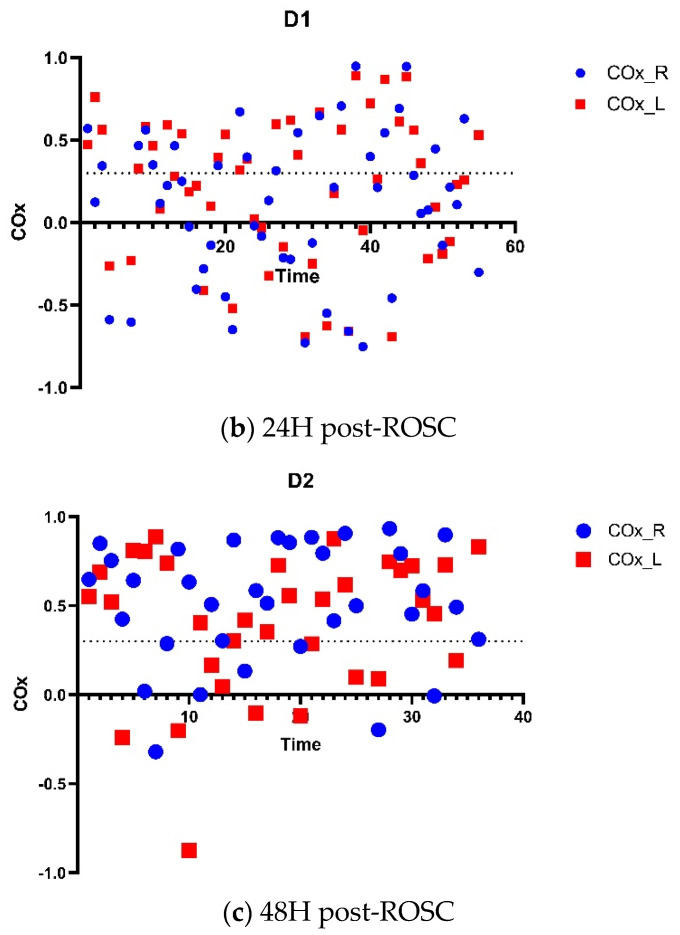
Cerebral autoregulation by NIRS. Cerebral autoregulation was assessed by NIRS on three consecutive days (**a**–**c**), starting from day of admission. The threshold of autoregulation was taken as CO_x_ of 0.3, represented by dotted lines. Threshold of autoregulation taken as CO_x_ 0.3, represented by the dotted line. The CO_x_ is a moving Pearson correlation coefficient between 30 consecutive, 10-s averaged values of MAP and the corresponding rSO_2_ signals. Majority of CO_x_ values were above 0.3 indicating impaired cerebral autoregulation. Abbreviations: NIRS, near-infrared spectroscopy; CO_x_, cerebral oximetry index; ROSC, return of spontaneous circulation; MAP, mean arterial pressure; rSO_2_, regional cerebral oxygenation.

Bedside TCD assessment and evaluation of cerebral autoregulation was performed during the first 3 consecutive days of admission. There was intact dynamic cerebral autoregulation based on Mx throughout the 3 days of monitoring (Figure 5). Hourly rSO_2_ readings showed initial dip in the first 6 h followed by steady recovery (Figure 2). Using CO_x_ of 0.3 as the threshold for loss of cerebral autoregulation, findings were less concordant with NIRS showing equivocal cerebral autoregulation on the second and third days (Figure 6). Plasma levels of NF-L, NF-H and suPAR were slightly elevated on admission, with temporal trends shown in Figure 2. Clinically, there was good neurological recovery and he was successfully extubated on D5. After 16 days of hospitalization (of which 9 days were spent in CCU), he was discharged home with minimal physical and cognitive deficits. 

### 3.4. Patient D

A 73-year-old Chinese man suffered an unwitnessed OHCA at home; he had complained of chest pain for at least 12 h prior to this. He did not receive bystander CPR. The initial rhythm documented by EMS was PEA which converted to VF in ED. Sustained ROSC was achieved after a downtime of 50 min. ECG post-ROSC showed anterior ST-segment elevations. Emergency coronary angiography showed 100% thrombotic occlusion of the proximal left anterior descending artery; coronary stenting was performed and IABP was inserted for cardiogenic shock. 

**Figure 7:**
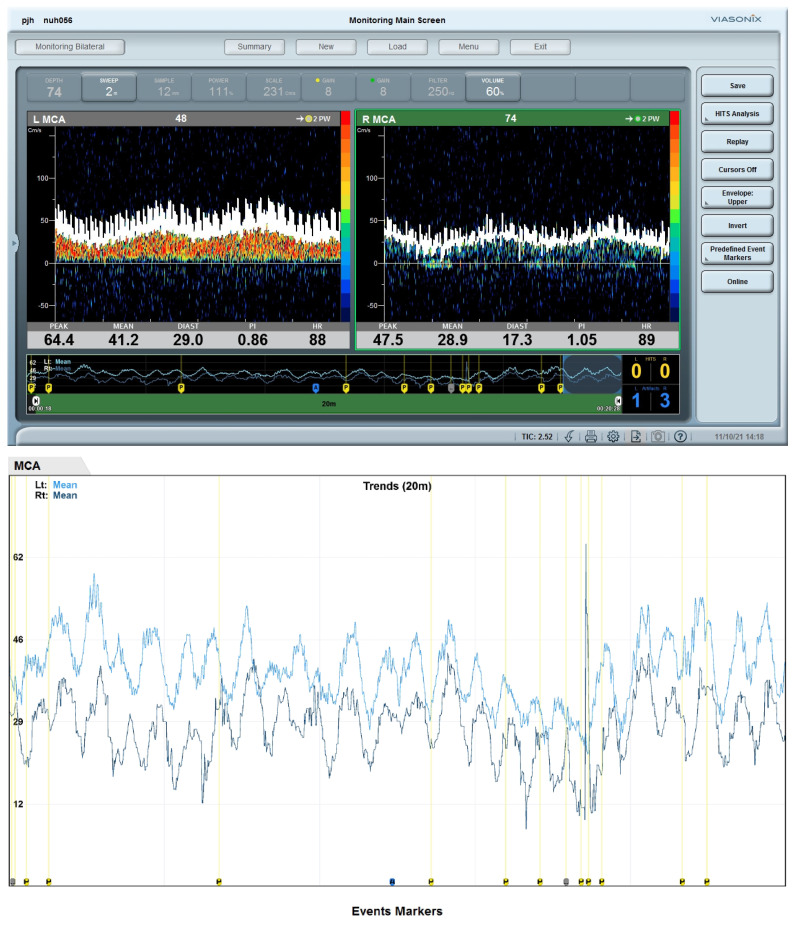
Cardio-cerebral asynchrony. The (**upper panel)** shows the screenshots of MFVs of both MCAs. The (**lower panel)** shows continuous recordings of the trends of MFV over a period of 20 min. Both MCAs show stereotypical cyclical changes in MFVs, which were independent of MAP, intervention with changing the dose of noradrenaline infusion, ETCO_2_ levels. This can be considered as cardio-cerebral asynchrony (or de-coupling) where MAP (cardio) and MFV in MCAs (cerebral) showed autonomous cyclical stereotypic fluctuations, independent of each other. Abbreviations: MFV, mean flow velocity; MCA, middle cerebral artery; MAP, mean arterial pressure; ETCO_2_, end-tidal carbon dioxide.

Bilateral rSO_2_ readings in the first 72 h were consistent with phasic variations in cerebral blood flow after cardiac arrest (Figure 2). Neuromonitoring with TCD was performed only on D5 after removal of IABP; by then, he had completed rSO_2_ monitoring and the sensor pads had been removed. TCD showed rhythmic variation in the mean CBFV of the left MCA (right MCA could not be monitored due to a suboptimal temporal acoustic window), with cycles of about 3 min, where CBFV increased independently of the changes in end-tidal carbon dioxide levels and MAP, remained elevated for about 2 min and returned back to the baseline (Figure 7). Plasma levels of NF-L, NF-H and suPAR increased significantly over time, particularly NF-L which showed a 900-fold increase over 72 h (Figure 2). Examination on D6 revealed poor GCS, dysconjugate gaze and absent reflexes. CT Brain showed generalized loss of grey-white matter differentiation in the supratentorial brain with mild sulci effacement, consistent with global hypoxic-ischemic injury. Electroencephalogram showed generalized encephalopathy. The patient’s stay in the CCU was complicated by further episodes of VF arrests, and hospital-acquired infection. He eventually succumbed to mixed septic and cardiogenic shock after 19 days of CCU stay.

## 4. Discussion

Our observational case series demonstrates the feasibility of real-time bedside assessments of cerebral autoregulation with concurrent TCD and NIRS with minimal interference of clinical care. Through continuous rSO_2_ monitoring and serial assessments of dynamic cerebral autoregulation in the first 72 h post-ROSC, we demonstrated phasic variation in cerebral blood flow velocities and observed that preserved cerebral autoregulation was associated with lower levels of neuro-injury biomarkers (NF-L and NF-H) and better neurological recovery at the time of hospital discharge. Interestingly, four different patterns of cerebral autoregulation and evolution of cerebral autoregulation were noted. 

Cerebral circulatory arrest, where alternating (reverberating) Doppler spectra were visualized in bilateral MCAs and basilar artery, was observed in a patient within 10 h of ROSC. Although cerebral circulatory arrest demonstrated by TCD is only considered as a supplementary test for brain death certification, it represents a highly specific marker of dismal neurological outcome, and has been shown to precede clinical brain death by 6–40 h [23]. We believe that such TCD findings may be considered as a tool for early neuroprognostication. 

Cardio-cerebral asynchrony, where cyclical changes in TCD-derived CBFV occurred independently of MAP, is suggestive of neurovascular uncoupling. Interestingly, PI as well as end-tidal carbon dioxide levels did not change during these cycles, suggesting impaired cerebral autoregulation. A similar pattern of MAP, not time-locked with CBFV changes, was noted, again independent of the dose of vasopressors. It is difficult to explain the underlying mechanism of such cardio-cerebral asynchrony or cardio-cerebral disconnection phenomena.

Our previous work on rSO_2_ monitoring in resuscitated cardiac arrest patients demonstrated phasic variations over the first 72 h, aligned with known variations in cerebral blood flow [24]. This, coupled with the varying cerebral hemodynamics and autoregulation demonstrated by TCD, may explain the neutral RCTs comparing static blood pressure and PaCO_2_ targets. We also observed discordant findings between TCD and NIRS in some patients during assessments of dynamic cerebral autoregulation, which may be explained by the following: (1) Signal contamination by scalp blood flow, ambient light or non-adherence of the monitoring pads to skin may result in artifacts in the NIRS signals. (2) The assumptions on which the accuracy of NIRS-derived rSO_2_ and cerebral autoregulation are contingent, such as constant arterial-venous ratio of frontal lobe microvasculature and cerebral oxygen metabolism, were not met in resuscitated OHCA patients. (3) Good TCD spectra is contingent on an experienced operator. More studies integrating real-time TCD, rSO_2_ and MAP are needed to allow us to compare the performance of NIRS against TCD in assessing dynamic cerebral autoregulation in patients with ROSC. 

Biomarkers are a key component of cardiac arrest prognostication algorithms with neuron-specific enolase (NSE) the only one integrated in contemporary clinical guidelines [2,3,25]. Its sensitivity to false positives and the lack of a standard assay has led to ongoing efforts to identify more accurate neuro-injury biomarkers. Neurofilaments (structural scaffolding proteins exclusively expressed in neurons) and suPAR (marker of immune activation and inflammation) have been shown to have varying performances as potential biomarkers of neuroprognostication, with neurofilament light (NF-L) demonstrating the best accuracy [26,27,28]. Although our case series showed significantly different temporal trends of NF-L, NFH and suPAR between those with good and poor neurological outcomes, our study was underpowered and not designed to evaluate the prognostic value of these biomarkers. A larger, adequately powered study is required to assess the predictive performance and define cut-offs for these biomarkers.

The major strength of our case series is the comprehensive multi-modal neuromonitoring in the first 72 h after ROSC, during which the concurrent use of TCD and NIRS allowed us to collect complementary cerebral macro- and microvascular information, which was further bolstered by biomarker data. These patients were part of the national OHCA database with comprehensive pre-hospital data collection based on Utstein definitions for reporting cardiac arrest [29]. Nonetheless, our study should be interpreted in the context of some limitations. While we were able to initiate continuous rSO_2_ monitoring in the early post-ROSC phase and standardize the duration of monitoring by leveraging on a protocol, TCD was performed at variable timepoints, with each assessment lasting 30–45 min. We missed the critical first 12 h post-ROSC in most patients as well as the evolution of cerebral blood flow in some patients in which TCD was not repeated due to logistic reasons. The main vasopressor used, if any, was noradrenaline and we assumed there was minimal effect of noradrenaline on cerebral blood flow. We lacked granular data on cardiac function and were unable to assess the impact of cardiac function on cerebral autoregulation, if any. Certain conditions, such as the presence of IABP, precluded TCD assessments. There was little to guide management of discordant TCD and rSO_2_ findings. 

Our study findings have important clinical implications. Having a protocol for early initiation of and serial multi-modal neuromonitoring with TCD and NIRS would prevent us from missing data in the critical early post-resuscitative phases. Capturing and integrating these multi-modality data on a single platform, with standard methods to determine and report cerebral autoregulation and cerebral autoregulation-based optimal MAP, would allow us to interpret and compare the data meaningfully. The between- and within-patient heterogeneity underscores the importance of individualizing treatment strategies in the early and intermediate phases after ROSC; this may explain the neutral findings in prior trials that utilized fixed (static) MAP and PaCO2 targets. An ongoing randomized controlled trial to determine if individualizing hemodynamic strategy targeting cerebral autoregulation-based optimal MAP improves clinical outcomes will provide more answers (NCT 05679739) on the feasibility and benefit of individualized treatment strategies in resuscitated OHCA patients. 

## 5. Conclusions

Our case series demonstrated the feasibility of bedside multi-modal assessment of cerebral autoregulation in resuscitated cardiac arrest patients. The between- and within-patient heterogeneity in cerebral blood flow calls for more research on individualized treatment strategies in the critical early and intermediate post-ROSC phases. Discordant NIRS and TCD findings call for more research in the integration of multi-modal neuromonitoring findings in order to identify phenotypes and interventions for neuroprotection in resuscitated cardiac arrest patients. 

## Figures and Tables

**Table 1:** Baseline characteristics.

Patient	Age	Smoker	HT	DM	CKD	CVD	Location	Initial Rhythm	Witnessed	First CPR	pH	Lactate	PCI	Cerebral Hemodynamics	Survived
1 (D)	73						Home	PEA		EMS	7.11	7.8	✓	No autoregulation	
2	74		✓		✓		Home	VT/VF	✓	Bystander	6.9	9.4		No autoregulation	
3 *	61	Unknown	✓	✓	✓		Home	PEA		Medical	6.76	1.4		Cerebral circulatory arrest	
4 (B) **	69		✓	✓	✓	✓	Home	Asystole		Medical	7.22	11.4		Cerebral circulatory arrest	
5	69		✓	✓		✓	Public	VT/VF	✓	Bystander	7.23	3.9		Preserved autoregulation	✓
6 (A)	65	Unknown	✓				Home	VT/VF		Bystander	7.13	10.3	✓	No autoregulation	
7	51	✓					Public	VT/VF	✓	EMS	7.21	3.9	✓	Preserved autoregulation	✓
8 (C)	45	✓	✓				Public	VT/VF	✓	EMS	7.23	2.7	✓	Preserved autoregulation	✓
9	76			✓	✓	✓	Medical	PEA	✓	Medical	6.95	>13.3	✓	Preserved autoregulation	
10	59	✓	✓			✓	Home	VT/VF	✓	Bystander	7.18	11.5	✓	Preserved autoregulation	✓
11	44	✓	✓	✓	✓	✓	Medical	VT/VF	✓	Medical	7.45	9.6		1: No autoregulation 2: Cerebral edema; preserved autoregulation 3: Preserved autoregulation	✓
12	47	✓					Home	VT/VF	✓	Bystander	7.16	2.7	✓	TCD aborted	
13	61	Ex-smoker	✓	✓		✓	Public	VT/VF	✓	EMS	7.11	7.4	✓	No autoregulation	
14	59	✓	✓	✓		✓	Public	Asystole	✓	EMS	7.29	7.8	✓	No autoregulation	
15 ***	69	✓				✓	Medical	Asystole	✓	Medical	7.22	1.1	✓	Preserved autoregulation	✓
16	59	✓	✓	✓			Home	Asystole	✓	EMS	7.37	12.8		Preserved autoregulation	✓

Table 1 depicts the baseline characteristics of adult resuscitated cardiac arrest patients. Patients (A) to (D) are described in the manuscript. Age refers to the age of patients in years at time of event. The first pH and lactate recorded after return of spontaneous circulation were used. All patients were independent in their activities of daily living prior to the cardiac arrest event, except one who had unknown pre-morbid status (*). All were male patients except one (**). All patients underwent emergency coronary angiogram. All except one (***) received TTM. All 7 survivors had good neurological recovery, defined as CPC of 1 or 2. Abbreviations: HT, hypertension; DM, diabetes mellitus; CKD, chronic kidney disease; CVD, cardiovascular disease; CPR, cardiopulmonary resuscitation; PCI, percutaneous coronary intervention; PEA, pulseless electrical activity; VT, ventricular tachycardia; VF, ventricular fibrillation; EMS, Emergency Medical Services; TTM, targeted temperature management; CPC, Cerebral Performance Category.

## Data Availability

The data used for this study are available from the corresponding author on reasonable request.
